# Fungal Prosthetic Valve Endocarditis by *Candida parapsilosis*: A Case Report

**DOI:** 10.5812/jjm.9428

**Published:** 2014-03-01

**Authors:** Tahereh Shokohi, Seyed Mahmood Nouraei, Mohammad Hosein Afsarian, Narges Najafi, Shirin Mehdipour

**Affiliations:** 1 Department of Parasitology and Mycology, Mazandaran University of Medical Sciences, Sari, IR Iran; 2 Invasive Fungi Research center, Mazandaran University of Medical Sciences, Sari, IR Iran; 3 Mazandaran Heart Center, Mazandaran University of Medical Sciences, Sari, IR Iran; 4 North Infectious Disease Research Center, Mazandaran University of Medical Sciences, Sari, IR Iran

**Keywords:** *Candida*, Fungi, Endocarditis, Prosthetic valve

## Abstract

**Introduction:**

Fungal prosthetic valve endocarditis (PVE) is rare but serious complication of valve replacement surgery. *Candida* species, particularly *Candida albicans* is the most common isolated pathogen in fungal PVE (1–6%of cases).

**Case Presentation:**

We describe a 35-year-old woman who underwent mechanical mitral valve replacement about 3 years ago. She was admitted with neurological symptoms and later with dyspnea and hypotension. Transesophageal echocardiography showed large and mobile prosthetic valve vegetation. She underwent mitral valve surgery. The explanted valve and vegetation revealed lots of budding yeasts and the isolated yeast was identified as *C. parapsilosis*. Amphotericin B and broad spectrum antibiotic were started immediately. Unfortunately, the patient died two days after surgery, due to sepsis probably related to the candidemia.

**Conclusions:**

Fungal endocarditis is uncommon infection, but it is a serious problem in patients with prosthetic valve. Fungal PVE can occur years after the surgery, thus long-term follow-up is essential.

## 1. Introduction

Fungal endocarditis (FE) is an uncommon infection (1, 2), and the prosthetic valve endocarditis (PVE) is a serious complication of valve replacement surgery (3). Its estimated incidence is 2.9% over the past two decades (4), and mortality rate, ranges from 23 to 46% (5-8). With increasing number of patients, who undergo prosthetic heart valve implantation, more individuals are at risk of PVE. Fungi, most commonly *Candida *species, are the causative pathogen in 2-10% of PVE cases (6). *Candida parapsilosis* is the second most common etiologic agent of fungal endocarditis. The prosthetic valve likely acted as the surface for developing fungal biofilms, which improve the antifungal resistance (9). Recent advances in medical and surgical treatments including reconstructive cardiovascular surgeries, implantation of intracardiac prosthetic devices, prolonged use of intravenous (IV) catheter, use of broad-spectrum antibiotics and immunosuppression drugs have been implicated as a cause for the rising number of fungemia and fungal endocarditis cases during the past two decades (10, 11).

## 2. Case Presentation

A 35-year-old woman, who underwent mechanical mitral valve replacement (31-mm St. Jude mechanical valve; ATS Medical Inc.) for sever valve regurgitation on 15th of July 2005. She took warfarin therapy with routine International Normalized Ratio (INR) check. In 29th of October 2008, she was admitted in Boo Ali Sina Teaching Hospital with neurological symptoms compatible with encephalitis. Complete blood count (CBC) showed hemoglobin level of 8.2 g/dL, WBC count of 5.9×10^9^ cells/L (80% polymorphonuclear leukocytes, 18% lymphocytes, and 2% monocytes), raised Erythrocyte sedimentation rate (ESR) and C-reactive protein (CRP 3+). Transthoracic echocardiography (TTE) showed no evidence of endocarditis. She was treated with ceftriaxone, metronidazole for a week and discharged in good health condition.

Thirty-five days later, she was readmitted to Razi Teaching Hospital with high temperature, malaise, cough, dyspnea, and hypotension. TTE showed arterial fibrillation with fast ventricular response and increased transprosthetic mitral valve gradient. Ceftriaxone, Vancomycin, Cefazolin, Gentamycin, and Warfarin were administered. She was then transferred to Mazandaran Heart Center for further evaluation. Transesophageal echocardiography showed a large and mobile prosthetic valve vegetation. She underwent mitral valve surgery on December 1, 2008. Under general anesthesia the chest was opened via median sternotomy. On cardio-pulmonary bypass left atrium was opened, white and creamy vegetations were all over and under the prosthetic valve. She underwent mitral valve replacement with 29-mm St. Jude mechanical valve. The explanted valve and vegetation were sent to laboratory for further evaluations. 

Direct microscopy (KOH 20%) and staining of vegetation showed lots of budding yeasts. Yeast colonies were yielded on Sabouraud Dextrose Agar (SDA; Oxoid, Wesel, Germany), after a 24-hour incubation at 27ºC. Using physiological and biochemical methods such as germ tube formation in horse serum, chlamydospore formation in CMA-Tween 80, colored colony on CHROMagar Candida medium (CHROMagar Company, France), API 20C Aux ( bioMerieux, France) strain was identified. The isolated microorganism was identified as *C. parapsilosis*. Amphotericin B and broad spectrum antibiotic were administered via peripherally inserted venous catheter. Prothrombin time (40s) and serum creatinine level (560 mg/d) were increased. The patient died two days after her surgery due to sepsis probably related to the candidemia. The patient was not clinically suspected to fungal PVE and the infection was diagnosed after surgery.

## 3. Conclusions

Fungal PVE is a rare but serious complication of valve replacement surgeries (3). *Candida* species, particularly *C. albicans* is the most common isolated pathogen in fungal PVE (1–6% of cases) (2). Most commonly, *Candida *endocarditis involves in the aortic and mitral valves (12). *Aspergillus *spp, is the second most frequent reported causative agent of fungal endocarditis (13). Prosthetic valve is an important risk factor for development of fungal endocarditis. Other predisposing factors are long-term intravenous catheters, broad-spectrum antibiotics, immunosuppression, pacemaker implantation and prior and concomitant bacterial endocarditis. The role of nosocomial fungemia in pathogenesis of fungal PVE is poorly understood (3). Nosocomial candidemia comprises about 10% of all bloodstream infections (14) and *Candida* species is the second cause of PVE, after the coagulase-negative staphylococci (15).

*Candida* species are the normal flora of GI tract, skin and oropharynx. Long term prophylactic antibiotic in open heart surgery and after prosthetic valve implantation separately or in conjunction with above mentioned factors make them susceptible to *Candida *endocarditis. The diagnosis of fungal PVE is extremely difficult because its clinical manifestations are identical to bacterial endocarditis. In a review of 91 patients with *Candida *endocarditis, 77% of them were diagnosed after the autopsy (16). Early diagnosis and treatment are crucial for the patients’ survival. As blood culture is negative in up to 50% of cases, TEE are the accepted modalities for detection of prosthetic valve vegetation with 60% sensitivity and 100% specificity (13).

We detected the fungal PVE by direct microscopy and culture of the valvular vegetation explanted prosthetic valve at the time of surgery. We could not confirm prior fungemia due to insufficient clinical and microbiologic data on the initial episode. With regards to the valve type, Calderwood et al. (5) reported threefold higher risk of PVE in early months of the surgery among patients with mechanical valve in comparison to those with bioprosthetic valve; however, the recipient of bioprosthetic had a higher risk of infection after 12 months or more after surgery. Rutledge et al. (17) found no significant difference in incidence of PVE in patient with mechanical and bioprosthetic valve. In our case, fungal PVE occurred three years after the surgery and was the late consequence. Although the fungal endocarditis is an uncommon disorder, but is a serious problem in patients with prosthetic valve. Hence, fungal PVE can occur years after surgery, thus long-term follow-up is essential.

## References

[1] Bayer AS, Scheld M, Mandell GL, Bennett JE, Dolin R (2000). Endocarditis and intravascular infections.. Principles and Practice of Infectious Diseases...

[2] Karchmer AW, Mandell GL, Bennett JE, Dolin R (2000). Infections on prosthetic valves and intravascular, devices.. Principles and Practice of Infectious Diseases...

[3] Melgar GR, Nasser RM, Gordon SM, Lytle BW, Keys TF, Longworth DL (1997). Fungal prosthetic valve endocarditis in 16 patients. An 11-year experience in a tertiary care hospital.. Medicine (Baltimore)..

[4] Threlkeld MG, Cobbs CG, Mandell GL, Bennett JE, Dolin R (1995). Infectious disorders of prosthetic valves and intravascular devices.. Principles and practice of infectious diseases..

[5] Calderwood SB, Swinski LA, Karchmer AW, Waternaux CM, Buckley MJ (1986). Prosthetic valve endocarditis. Analysis of factors affecting outcome of therapy.. J Thorac Cardiovasc Surg..

[6] Fang G, Keys TF, Gentry LO, Harris AA, Rivera N, Getz K (1993). Prosthetic valve endocarditis resulting from nosocomial bacteremia. A prospective, multicenter study.. Ann Intern Med..

[7] Grover FL, Cohen DJ, Oprian C, Henderson WG, Sethi G, Hammermeister KE (1994). Determinants of the occurrence of and survival from prosthetic valve endocarditis. Experience of the Veterans Affairs Cooperative Study on Valvular Heart Disease.. J Thorac Cardiovasc Surg..

[8] Sett SS, Hudon MP, Jamieson WR, Chow AW (1993). Prosthetic valve endocarditis. Experience with porcine bioprostheses.. J Thorac Cardiovasc Surg..

[9] Kumar J, Fish D, Burger H, Weiser B, Ross JS, Jones D (2011). Successful surgical intervention for the management of endocarditis due to multidrug resistant Candida parapsilosis: case report and literature review.. Mycopathologia..

[10] Fernandez-Guerrero ML, Verdejo C, Azofra J, de Gorgolas M (1995). Hospital-acquired infectious endocarditis not associated with cardiac surgery: an emerging problem.. Clin Infect Dis..

[11] Rubinstein E, Lang R (1995). Fungal endocarditis.. Eur Heart J..

[12] Muehrcke DD, Lytle BW, Cosgrove DM, 3rd (1995). Surgical and long-term antifungal therapy for fungal prosthetic valve endocarditis.. Ann Thorac Surg..

[13] Nguyen MH, Nguyen ML, Yu VL, McMahon D, Keys TF, Amidi M (1996). Candida prosthetic valve endocarditis: prospective study of six cases and review of the literature.. Clin Infect Dis..

[14] Pfaller MA (1994). Epidemiology and control of fungal infections.. Clin Infect Dis..

[15] Banerjee SN, Emori TG, Culver DH, Gaynes RP, Jarvis WR, Horan T (1991). Secular trends in nosocomial primary bloodstream infections in the United States, 1980-1989. National Nosocomial Infections Surveillance System.. Am J Med..

[16] Seelig MS, Speth CP, Kozinn PJ, Taschdjian CL, Toni EF, Goldberg P (1974). Patterns of Candida endocarditis following cardiac surgery: Importance of early diagnosis and therapy (an analysis of 91 cases).. Prog Cardiovasc Dis..

[17] Rutledge R, Kim BJ, Applebaum RE (1985). Actuarial analysis of the risk of prosthetic valve endocarditis in 1,598 patients with mechanical and bioprosthetic valves.. Arch Surg..

